# Septic Abortion Complicated by Disseminated Intravascular Coagulation

**DOI:** 10.21980/J8GH1G

**Published:** 2024-04-30

**Authors:** Lauren Moore, Jennifer Yee

**Affiliations:** * The Ohio State University Wexner Medical Center, Department of Emergency Medicine, Columbus, OH

## Abstract

**Audience:**

This scenario was developed to educate emergency medicine residents on the diagnosis and management of two concurrent conditions: septic abortion and disseminated intravascular coagulation (DIC).

**Introduction:**

Patients with an abortion (spontaneous or induced) of less than twenty weeks gestation may present with concurrent uterine infection, also known as septic abortion. One of the complications of septic abortion is DIC. Early management of both underlying etiology (septic abortion) and subsequent complications (DIC) is crucial to minimize morbidity and mortality.

**Educational Objectives:**

At the conclusion of the simulation session, learners will be able to: 1) Obtain a relevant focused history including pregnancy history, medication use, and past medical history. 2) Develop a differential for fever and vaginal bleeding in a pregnant patient. 3) Discuss management of septic abortion, including empiric broad-spectrum antibiotics and obstetric consultation for source control with dilation and curettage (D&C). 4) Discuss expected laboratory findings of disseminated intravascular coagulation (DIC). 5) Discuss management of DIC, including identification of underlying etiology and supportive resuscitation with blood products. 6) Review the components of blood products. 7) Identify appropriate disposition of the patient to the intensive care unit (ICU).

**Educational Methods:**

This session was conducted using high-fidelity simulation followed by a debriefing session and discussion about the diagnosis, differential, and management of both septic abortion and DIC. Debriefing methods may be left to the discretion of participants, but the authors have utilized advocacy-inquiry techniques. In this technique, the facilitator described something they observed in the case, outlined their reasoning as a facilitator why this observation was important or why they had questions, and then asked the learners to share their frame of reference at the time. An example: “I heard the team leader state that the platelets were normal, but then another resident disagreed. No one paused to come to a consensus. I’m wondering why this wasn’t explored further in real time. Tell me more.” This scenario may also be run as an oral boards case or adapted for other learners such as critical care fellows.

**Research Methods:**

Our residents were provided a survey at the completion of the debriefing session so they could rate different aspects of the simulation, as well as provide qualitative feedback on the scenario. The local institution’s simulation center’s electronic feedback form is based on the Center of Medical Simulation’s Debriefing Assessment for Simulation in Healthcare (DASH) Student Version Short Form,^1^ with the inclusion of required qualitative feedback if an element was scored less than a 6 or 7.

**Results:**

Thirteen learners completed a feedback form out of seventeen participants. This session received all six and seven scores (consistently effective/very good and extremely effective/outstanding, respectively) other than two isolated 4 scores.

**Discussion:**

This is a cost-effective method for reviewing septic abortion and DIC. The case may be modified for appropriate audiences, such as simplifying the case to septic abortion without DIC. You can also consider not showing an initial temperature with the initial set of vitals unless it is specifically asked for by the participants. We encourage readers to utilize bleeding moulage techniques as a visual stimulus to increase psychological buy-in.

**Topics:**

Medical simulation, septic abortion, pregnancy complications, hematology emergencies, obstetric emergencies, disseminated intravascular coagulation, emergency medicine.

## USER GUIDE

[Table t1-jetem-9-2-s1]

**Table t1-jetem-9-2-s1:** 

List of Resources:	
Abstract	1
User Guide	3
Instructor Materials	5
Operator Materials	15
Debriefing and Evaluation Pearls	17
Simulation Assessment	22

**Learner Audience:**

Interns, Junior Residents, Senior Residents

**Time Required for Implementation:**

Instructor Preparation: 30 minutes

Time for case: 20 minutes

Time for debriefing: 40 minutes

**Recommended Number of Learners per Instructor:**

3–4

**Topics:**

Medical simulation, septic abortion, pregnancy complications, hematology emergencies, obstetric emergencies, disseminated intravascular coagulation, emergency medicine.

**Objectives:**

By the end of this simulation session, the learner will be able to:

1. Obtain a relevant focused history, including pregnancy history, medication use, and past medical history.
2. Develop a differential for fever and vaginal bleeding in a pregnant patient.
3. Discuss management of septic abortion, including empiric broad spectrum antibiotics and obstetric consultation for source control with dilation and curettage (D&C).
4. Discuss expected laboratory findings of disseminated intravascular coagulation (DIC).
5. Discuss management of DIC, including identification of underlying etiology and supportive resuscitation with blood products.
6. Review the components of blood products.
7. Identify appropriate disposition of the patient to the intensive care unit (ICU).

### Linked objectives, methods and results

Patients with septic abortion require prompt recognition, treatment, and transfer to an operating room for D&C once initially stabilized. In this scenario, providers will review how to clinically diagnose septic abortion related to retained products of conception with an appropriately focused history and physical exam (objective 1) while still considering and ruling out other differentials that may present similarly (objective 2). Participants should recognize the importance of early resuscitation of sepsis with intravenous fluids and administer broad-spectrum antibiotics (objective 3). Labs and blood cultures should be promptly obtained. In this scenario, participants will also review that DIC may complicate the clinical picture and that their treatment regimen must be expanded to address the need of blood product transfusion (objectives 4, 5, 6). Once the patient has been resuscitated, she should be taken to the operating room (OR) by obstetrics-gynecology (OB-GYN) for a D&C (objective 3) before being admitted in the ICU for further management (objective 7). This simulation scenario allows learners to reinforce septic abortion and DIC management skills in a psychologically-safe learning environment, and then receive formative feedback on their performance.

### Recommended pre-reading for instructor

- We recommend that instructors review literature regarding septic abortion and disseminated intravascular coagulation (DIC), including presenting signs/symptoms, diagnosis, and management. Suggested readings include materials listed under the “References/suggestions for further reading” section below.

### Results and tips for successful implementation

This simulation was written for emergency medicine residents to be performed as a high-fidelity simulation scenario. It also may be used as a mock oral board case, or as a case for learners in other specialties. We ran this case with nine small groups of emergency medicine residents over three days.

This session received all six and seven scores (consistently effective/very good and extremely effective/outstanding, respectively) other than two isolated 4 scores. The lowest average score was tied at 6.53 for “Before the simulation, the instructor set the stage for an engaging learning experience,” and “The instructor identified what I did well or poorly - and why.” The highest average score was tied at 6.84 for “During the simulation, the instructor maintained an engaging context for learning,” and “The instructor provoked in-depth discussions that led me to reflect on my performance.” The form also includes an area for general feedback about the case at the end. Illustrative examples of feedback include: “Always feel these are great thinking problems, love being in a low-risk situation first,” and “Great review of this less frequent presentation.”

1. **Before the simulation, the instructor set the stage for an engaging learning experience.** (scores 4–7; 4 comment: “came late.” Mean: 6.54, median: 7)
2. **During the simulation, the instructor maintained an engaging context for learning.** (scores 6–7. Mean: 6.85, median: 7)
3. **The instructor structured the debriefing in an organized way.** (scores 6–7. Mean: 6.77, median: 7)
4. **The instructor provoked in-depth discussions that led me to reflect on my performance.** (scores 6–7. Mean: 6.85, median: 7)
5. **The instructor identified what I did well or poorly - and why.** (scores 4–7; 4 comment: “No specific feedback.” Mean: 6.54, median: 7)
6. **The instructor helped me see how to improve or how to sustain good performance.** (scores 6–7. Mean: 6.77, median: 7)

One recurring theme that we noticed was that the learners didn’t notice the moulage placed at the intravenous (IV) catheter site to represent ongoing bleeding. Nursing often had to prompt the learners by pointing out the “blood.” In several instances, learners did not recognize this as a trigger to order DIC labs. In these cases, nursing would comment how the IV site was “requiring a lot of pressure to stop bleeding.” Alternatively, moulage depicting petechiae may be used as an additional visual prompt.

## Supplementary Information



## Figures and Tables

**Table t2-jetem-9-2-s1:** 

#1	Complete blood count (CBC)
#2	Basic metabolic panel (BMP)
#3	Liver function tests (LFTs)
#4	PTT
#5	PT/INR
#6	D-dimer
#7	Fibrinogen
#8	Human chorionic gonadotropin (hCG) qualitative
#9	hCG quantitative
#10	Lactate
#11	Venous blood gas
#12	Urinalysis
#13	Electrocardiogram
#14	Pelvic ultrasound
#15	Chest Radiograph (X-ray)

**Table t3-jetem-9-2-s1:** 

**Vitals:**	*Heart Rate (HR)* 125	*Blood Pressure (BP)* 90/60	*Respiratory Rate (RR)* 24
	*Temperature (Temp)* 103.0°F	*Oxygen Saturation (O*_*2*_*Sat)* 97% on room air	

**Table t4-jetem-9-2-s1:** 

*Complete blood count (CBC)*	
White blood count (WBC)	23.4 ×1000/mm^3^(H)
Hemoglobin (Hgb)	6.5 g/dL (L)
Hematocrit (HCT)	18.0 % (L)
Platelet (Plt)	45 x1000/mm^3^ (L)
Bands 17%	(H)

**Table t5-jetem-9-2-s1:** 

*Basic metabolic panel (BMP)*	
Sodium	138 mEq/L
Chloride	99 mEq/L
Potassium	4.5 mEq/L
Bicarbonate (HCO_3_)	14 mEq/L (L)
Blood Urea Nitrogen (BUN)	36 mg/dL
Creatinine (Cr)	1.2 mg/dL
Glucose	110 mg/dL
Calcium	8.0 mg/dL

**Table t6-jetem-9-2-s1:** 

*Liver Function Test (LFT)*	
Total bilirubin	2.8 mg/dL (H)
Direct bilirubin	0.2 mg/dL
Albumin	3.0 g/dL
Alkaline Phosphate	100 U/L
Aspartate Aminotransferase (AST)	30 u/L
Alanine Aminotransferase (ALT)	40 u/L

**Table t7-jetem-9-2-s1:** 

*Prothrombin time*	89 seconds (H)

**Table t8-jetem-9-2-s1:** 

*Activated partial thromboplastin time*	20 seconds
*International normalized ratio*	1.7 (H)

**Table t9-jetem-9-2-s1:** 

*Fibrinogen*	90 mg/dL (150–400mg/dL) (L)

**Table t10-jetem-9-2-s1:** 

*hCG qualitative*	Positive (!)

**Table t11-jetem-9-2-s1:** 

*hCG quantitative*	40,380 mIU/mL

**Table t12-jetem-9-2-s1:** 

*Lactate*	5.0 mEq/L (H)

**Table t13-jetem-9-2-s1:** 

*Venous blood gas (VBG)*	
pH	7.22 (L)
pCO2	25 mmHg (L)
pO2	63 mmHg
HCO3	14 mEq/L (L)
O_**2**_ Saturation	96% on room air

**Table t14-jetem-9-2-s1:** 

*Urinalysis*	
Color	Brown (!)
Spec gravity	1.015
Glucose	negative
Ketones	2+ (H)
Hemoglobin	large (H)
Protein	negative
Leukocyte esterase	negative
Nitrite	negative
Red blood cells (RBC)	0–5/HPF
White blood cells (WBC)	>50/HPF (H)
Squamous epithelial	10–15/HPF (H)

**Figure f1-jetem-9-2-s1:**
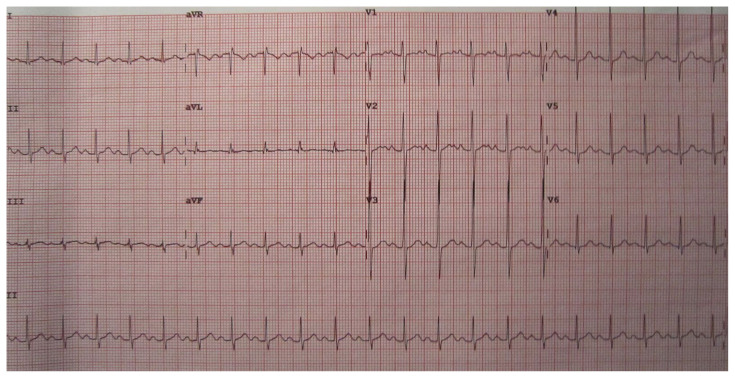


**Figure f2-jetem-9-2-s1:**
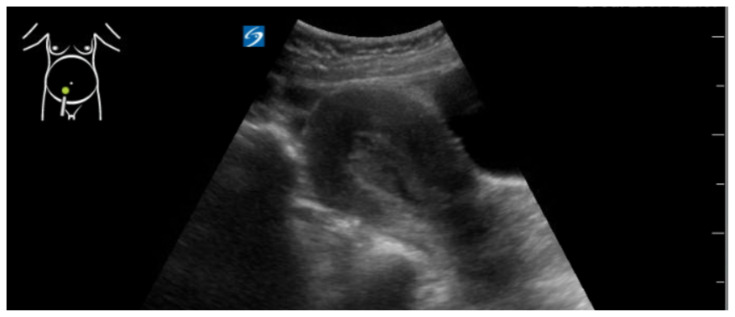


**Figure f3-jetem-9-2-s1:**
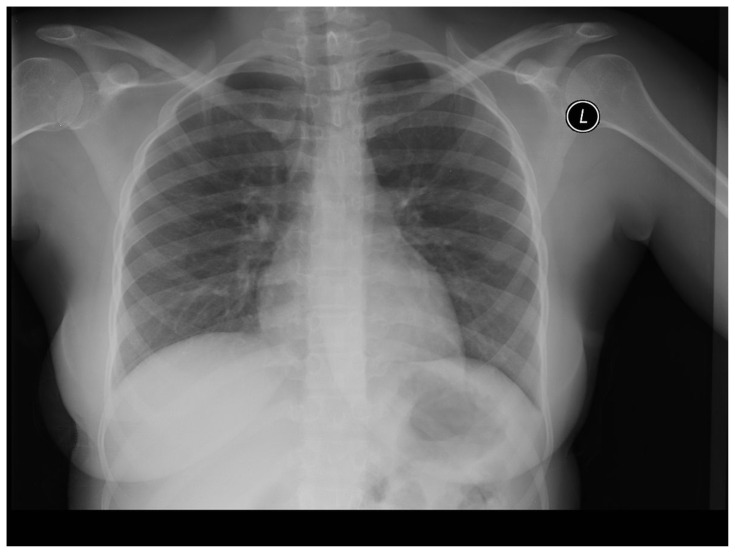


**Table t15-jetem-9-2-s1:** SIMULATION EVENTS TABLE:

Minute (state)	Participant action/trigger	Patient status (simulator response) & operator prompts	Monitor display (vital signs)
0:00 (Baseline)	Team enters patient’s room in the emergency department.	Participants should begin by placing the patient on a monitor, obtaining history from patient, and performing a physical exam. IV placed based upon abnormal VS.	A: T 103°F HR 125 BP 90/60 RR 24 O_2_ sat 98% room air
4:00	Team should recognize the patient is septic. IV placed, labs obtained. Labs, Blood cultures, UA, EKG, and CXR should be ordered.	If the team administers a 30 cc/kg bolus, tachycardia and hypotension will improve (B). If the team does not administer a 30 cc/kg bolus within the first 5 minutes, tachycardia and hypotension will worsen(C).	
8:00	Nurse places blood moulage surreptitiously at IV insertion site as team reviews laboratory findings, and then points the bleeding out to them. Team should recognize that patient is in DIC and give FFP, pRBCs and platelets.	Team should add on coagulation factors, fibrinogen, and d-dimer to labs once bleeding at IV site is noted. Treat fever with anti-pyretic. If team administers appropriate blood products including pRBC, platelets, AND FFP, then vitals improve (D). If appropriate blood products are not given by minute 10, tachycardia, hypotension, tachypnea worsen (E).	B: (GIVEN 30 cc/kg bolus) T 103°F HR 110 BP 105/70 RR 20 O_2_ sat 98% RA C: (NOT GIVEN 20 cc/kg bolus) T 103°F HR 140 BP 80/50 RR 30 O_2_ sat 92% RA
12:00	OB should be consulted for emergent D&C. Participants will contact admitting team for ICU admission following D&C for further resuscitation.	The team should consult OBGYN and advocate that the patient needs emergent D&C. If the team does not state that the patient needs a D&C, the OBGYN team could prompt “what do you think she needs from us?” If the team calls the ICU or any other team for admission instead of OBGYN, the admitting team could prompt that “it sounds like we need definitive care of the underlying cause. Call me back when that’s done.” If IVF resuscitation/antibiotics/blood products have not yet been given, the OBGYN team will ask for continued resuscitation prior to taking her to the OR. Learners can be prompted to give blood products or fluids. If the team does not offer that the patient will need critical care following the OR due to sepsis/DIC, the nurse should ask “what level of care will the patient need?”	D: (Given blood products, antibiotics, anti- pyretics, AND bolus) T 100.5°F HR 95 BP 120/75 RR 18 O_2_ sat 98% RA E: FFP, platelets, AND blood products NOT given by minute 10 T 101°F HR 165 BP 75/45 RR 32 O_2_ sat 92% RA

**Table t16-jetem-9-2-s1:** 

Blood Product	Packed Red Blood Cells (pRBC)	Platelets	Fresh Frozen Plasma (FFP) Contains all coagulation factors and fibrinogenn	Cryoprecipitate Is a precipitate of FFP and contains only fibrinogen, vWF, and factor VIII
**Goal of administration**	Increase oxygen carrying capacity	Decrease risk of bleeding	Decrease risk of bleeding	Replace fibrinogen
**1 bag equivalency**	1 unit = 350ml raises hemoglobin (Hgb) by approximately 1 point	1 pack = 5 units = 250ml = raises platelets by 250k	1 unit = 250ml at 10ml/kg raises factor level by 25%	5 units = 200ml
**Indication** (other than clinical judgment in active bleeding)	Hgb <7mg/dL	<50k with planned neurosurgical procedure or actively bleeding <20k for febrile patient or coagulopathy <10k in asymptomatic patients for prophylaxis	Warfarin reversal (if INR is >1.7), bleeding with coagulopathy and PT/PTT >1.5x normal	In massive hemorrhage with a fibrinogen level <100 and bleeding in von Willebrand disease unresponsive to treatment
**Notes**		Low quality of evidence related to indications		1 unit of FFP has the equivalent of only 2 units of cryoprecipitate

**Table t17-jetem-9-2-s1:** 

**Critical Actions** Place patient on monitor and obtain a full set of vitals, including temperature Place two large bore IV catheters Initiate administration of 30 cc/kg intravenous fluid bolus within three minutes of noting tachycardia Perform a physical exam specifically including a pelvic exam Order broad-spectrum antibiotics for appropriate polymicrobial coverage Communicate need for D&C for retained products of conception to OB-GYN Reassess patient when nursing staff notes bleeding from peripheral IV sites Order a d-dimer, fibrinogen, and coagulation factors Verbalize concern for DIC Administer packed red blood cells, fresh frozen plasma, and platelets in a 1:1:1 ratio. Admit to the ICU for further management of sepsis and bleeding	0:00

**Table t18-jetem-9-2-s1:** 

	Milestone	Did not achieve level 1	Level 1	Level 2	Level 3
1	**Emergency Stabilization (PC1)**	□ Did not achieve Level 1	□ Recognizes abnormal vital signs	□ Recognizes an unstable patient, requiring intervention Performs primary assessment Discerns data to formulate a diagnostic impression/plan	□ Manages and prioritizes critical actions in a critically ill patient Reassesses after implementing a stabilizing intervention
2	**Performance of focused history and physical (PC2)**	□ Did not achieve Level 1	□ Performs a reliable, comprehensive history and physical exam	□ Performs and communicates a focused history and physical exam based on chief complaint and urgent issues	□ Prioritizes essential components of history and physical exam given dynamic circumstances
3	**Diagnostic studies (PC3)**	□ Did not achieve Level 1	□ Determines the necessity of diagnostic studies	□ Orders appropriate diagnostic studies. Performs appropriate bedside diagnostic studies/procedures	□ Prioritizes essential testing Interprets results of diagnostic studies Reviews risks, benefits, contraindications, and alternatives to a diagnostic study or procedure
4	**Diagnosis (PC4)**	□ Did not achieve Level 1	□ Considers a list of potential diagnoses	□ Considers an appropriate list of potential diagnosis May or may not make correct diagnosis	□ Makes the appropriate diagnosis Considers other potential diagnoses, avoiding premature closure
5	**Pharmacotherapy (PC5)**	□ Did not achieve Level 1	□ Asks patient for drug allergies	□ Selects an medication for therapeutic intervention, consider potential adverse effects	□ Selects the most appropriate medication and understands mechanism of action, effect, and potential side effects Considers and recognizes drug-drug interactions
6	**Observation and reassessment (PC6)**	□ Did not achieve Level 1	□ Reevaluates patient at least one time during case	□ Reevaluates patient after most therapeutic interventions	□ Consistently evaluates the effectiveness of therapies at appropriate intervals
7	**Disposition (PC7)**	□ Did not achieve Level 1	□ Appropriately selects whether to admit or discharge the patient	□ Appropriately selects whether to admit or discharge Involves the expertise of some of the appropriate specialists	□ Educates the patient appropriately about their disposition Assigns patient to an appropriate level of care (ICU/Tele/Floor) Involves expertise of all appropriate specialists
9	**General Approach to Procedures (PC9)**	□ Did not achieve Level 1	□ Identifies pertinent anatomy and physiology for a procedure Uses appropriate Universal Precautions	□ Obtains informed consent Knows indications, contraindications, anatomic landmarks, equipment, anesthetic and procedural technique, and potential complications for common ED procedures	□ Determines a back-up strategy if initial attempts are unsuccessful Correctly interprets results of diagnostic procedure
20	**Professional Values (PROF1)**	□ Did not achieve Level 1	□ Demonstrates caring, honest behavior	□ Exhibits compassion, respect, sensitivity and responsiveness	□ Develops alternative care plans when patients’ personal beliefs and decisions preclude standard care
22	**Patient centered communication (ICS1)**	□ Did not achieve level 1	□ Establishes rapport and demonstrates empathy to patient (and family) Listens effectively	□ Elicits patient’s reason for seeking health care	□ Manages patient expectations in a manner that minimizes potential for stress, conflict, and misunderstanding. Effectively communicates with vulnerable populations, (at risk patients and families)
23	**Team management (ICS2)**	□ Did not achieve level 1	□ Recognizes other members of the patient care team during case (nurse, techs)	□ Communicates pertinent information to other healthcare colleagues	□ Communicates a clear, succinct, and appropriate handoff with specialists and other colleagues Communicates effectively with ancillary staff

## INSTRUCTOR MATERIALS

**Case Title:** Septic Abortion Complicated by Disseminated Intravascular Coagulation

**Case Description & Diagnosis (short synopsis):** The patient is a 21-year-old gravida (G)3, para (P)2012 female who presents with abdominal pain and vaginal bleeding.

Patient is now febrile and tachycardic. At this time, participants should complete a full physical examination during which they should appreciate lower abdominal/uterine tenderness and continued mild to moderate vaginal bleeding on pelvic exam without other significant abnormality. Initial laboratory workup demonstrates a positive pregnancy test as well as a leukocytosis, anemia, and thrombocytopenia. The team should have a low threshold to evaluate and treat sepsis, including cultures, antibiotics, and initial resuscitation with 30 milliliters per kilogram (cc/kg) of fluids. Two sets of blood cultures should be obtained.

After reviewing labs, nursing will point out that the patient is now oozing from her IV line sites. Participants should then order labs to assess for DIC, including hepatic function tests, coagulation studies, a d-dimer, and fibrinogen level.

Tachycardia and hypotension will initially improve with fluid resuscitation but will then decompensate until participants give fresh frozen plasma (FFP) to a repletion goal of prothrombin (PT)/partial thromboplastin time (PTT) <1.5x normal limit and platelets to goal of >50,000 in setting of active bleeding.

If participants request a pelvic ultrasound, which is not a critical action, it will demonstrate retained products of conception (RPOC). Regardless, the team will need to recognize based on the scenario that RPOC is the most likely underlying etiology for both conditions and place an emergent OB-GYN consult for D&C. OB-GYN will not evaluate the patient without adequate blood product resuscitation or without the team explicitly advocating for D&C. The patient will ultimately need further resuscitation and management in the ICU.

**Equipment or Props Needed:**

● High fidelity simulation mannequin with female genital insert or pelvic task trainer
● Bedside ultrasound machine, if available
● Angiocatheters for large bore peripheral intravenous access = 18 gauge, 20 gauge
● Cardiac monitor
● Pulse oximetry
● IV pole
● Speculum with water-soluble lubrication (if mannequin has ability to perform a pelvic exam or if pelvic task trainer is available)
● Personal protective equipment for participants, including gowns, gloves, and masks
● Blood moulage
● Normal saline (1 liter x2)
● Lactated Ringers (1 liter x2)
● Simulated medications with labeling: cefepime, piperacillin-tazobactam, vancomycin, metronidazole, clindamycin, ampicillin, gentamycin
● Simulated blood products: 2 units of packed red blood cells (pRBCs), 2 units of FFP, 2 unit of platelets, 2 units of cryoprecipitate

**Actors needed:**

Primary nurse

Faculty may call in overhead as the obstetrics-gynecology provider

**Stimulus Inventory:** [Table t2-jetem-9-2-s1]

**Background and brief information:** The confederate nurse states to the team upon arrival to the simulation bay: “Patient is a 21-year-old female who presents with vaginal bleeding.”

**Initial presentation:** 21-year-old female who appears to be in pain. She is protecting her airway without difficulty.

- Past medical history: none
- Past surgical history: right knee arthroscopy at 15 years old
- Past pregnancy history: G3P2012, last menstrual period (LMP) was 10 weeks ago. Children are 4 and 2 years old.
- Medications: none
- Allergies: none
- Social hx: social alcohol use, five pack/year cigarette smoker
- Family history: noncontributory
- Vital signs:
  ○ HR – 125 beats per minute
  ○ Resp rate – 24 respirations per minute
  ○ Temp – 103.0° Fahrenheit (F)
  ○ BP – 90/60 millimeters of mercury (mmHg)
  ○ Pulse ox – 98% on room air
- Weight: 65 kg
- Assessment: Lying supine, holding hands over her lower abdomen, appears uncomfortable

**How the scenario unfolds:** Participants should elicit more information from the patient including that five days ago the patient had an elective abortion at suspected 9–10 weeks of gestation by LMP and has been feeling worsening abdominal pain and vaginal bleeding since then. Today she felt weaker and had a fever to 103°F at home. A physical exam including a pelvic exam should be performed with vaginal bleeding and uterine tenderness noted.

During this time, the team should have a low threshold to evaluate and treat sepsis including cultures, antibiotics, and initial resuscitation with fluids. Once initial laboratory results are reviewed, nursing (confederate) will surreptitiously place blood at the intravenous line insertion site via a ten-milliliter syringe and point out the bleeding to the learners, who should then order labs to assess for acute DIC.

Patient will initially have improved vitals with fluid resuscitation but will decompensate until participants enact mass transfusion protocol and give packed red blood cells, FFP to repletion goal of PT/PTT <1.5x normal limit, and platelets to goal of >50,000 mm^3^ in setting of active bleeding. Most importantly, they will need to recognize that the patient has retained products of conception leading to sepsis and DIC, and will need to treat the underlying cause with antibiotics and an OB consult for D&C.

**Critical Actions:**

1. Place patient on monitor and obtain a full set of vitals, including temperature
2. Place two large bore IV catheters
3. Initiate administration of 30 cc/kg intravenous fluid bolus within three minutes of noting tachycardia
4. Perform a physical exam specifically including a pelvic exam
5. Order broad-spectrum antibiotics for appropriate polymicrobial coverage
6. Communicate need for D&C for retained products of conception to OB-GYN
7. Reassess patient when nursing staff notes bleeding from peripheral IV sites
8. Order a d-dimer, fibrinogen, and coagulation factors
9. Verbalize concern for DIC
10. Administer packed red blood cells, fresh frozen plasma, and platelets in a 1:1:1 ratio.
11. Admit to the ICU for further management of sepsis and bleeding

**Case Title:** Septic Abortion Complicated by Disseminated Intravascular Coagulation

**Chief Complaint:** Vaginal bleeding[Table t3-jetem-9-2-s1]

**General Appearance:** Lying supine, holding hands over her lower abdomen, appears uncomfortable.

**Primary Survey:**

- **Airway:** patent
- **Breathing:** clear to auscultation bilaterally
- **Circulation:** tachycardic, 2+ symmetric radial and dorsalis pedis pulses bilaterally, capillary refill >3 seconds

**History:**

- **History of present illness:** Unable to obtain much from the patient due to pain though she is able to state she has abdominal pain and is bleeding. If pregnancy history specifically elicited, the patient underwent an uncomplicated elective abortion five days ago when she was at approximately 9–10 weeks gestation by LMP. Since then, she has had worsening abdominal pain and vaginal bleeding but today developed a fever to 102°F at home and felt very weak. She is here alone.
- **Past medical history:** G3P2012
- **Past surgical history:** right knee arthroscopy at 15 years old
- **Patient’s medications:** none
- **Allergies:** none
- **Social history:** cigarette smoker 5 pack years, no alcohol or illicit drug use
- **Family history:** noncontributory
- **Weight:** 65 kg

**Secondary Survey/Physical Examination:**

- **General appearance:** lying supine with hands over lower abdomen looking uncomfortable, appears stated age
- **HEENT:**
  ○ **Head:** atraumatic
  ○ **Eyes:** pale conjunctiva, otherwise within normal limits
  ○ **Ears:** within normal limits.
  ○ **Nose:** within normal limits.
  ○ **Throat:** within normal limits.
- **Neck**: within normal limits.
- **Heart**: regular rhythm, tachycardic. Otherwise within normal limits
- **Lungs**: clear to auscultation bilaterally. Mild tachypnea with conversation. Otherwise within normal limits
- **Abdominal/GI**: soft abdomen. Moderate to significant suprapubic tenderness to palpation with guarding but without rebound. No distention or rigidity
- **Genitourinary:** bright red blood on her inner thighs and vulva. Closed os with dark red blood pooling in the vaginal vault with active oozing of blood. Tenderness to palpation over the uterus
- **Rectal**: digital rectal exam deferred. No bleeding seen externally
- **Extremities**: one 20-gauge peripheral in right antecubital fossa that is oozing at the insertion site. Otherwise within normal limits
- **Neuro**: within normal limits
- **Skin**: dry, capillary refill reduced to >4 seconds. Oozing blood at peripheral IV line site once placed by nursing after initial laboratory findings reviewed
- **Lymph**: within normal limits
- **Psych**: within normal limits

**Results:** [Table t4-jetem-9-2-s1] [Table t5-jetem-9-2-s1] [Table t6-jetem-9-2-s1] [Table t7-jetem-9-2-s1] [Table t8-jetem-9-2-s1] [Table t9-jetem-9-2-s1] [Table t10-jetem-9-2-s1] [Table t11-jetem-9-2-s1] [Table t12-jetem-9-2-s1] [Table t13-jetem-9-2-s1] [Table t14-jetem-9-2-s1]

*Electrocardiogram*

Heilman J. Sinus tachycardia as seen on ECG. In: Wikimedia Commons.

https://commons.wikimedia.org/wiki/File:Sinustachy.JPG. Published June 15, 2012. CC BY-SA 3.0.[Fig f1-jetem-9-2-s1]

*Pelvic ultrasound*

Author’s own image demonstrating retained products of conception.[Fig f2-jetem-9-2-s1]

*Chest X-ray*

Rosen Y. Normal PA chest x-ray. In: Wikimedia Commons.

https://commons.wikimedia.org/wiki/File:Normal_PA_chest_x-ray_(5414485536).jpg. Published 3 February 2011. Public domain.[Fig f3-jetem-9-2-s1]

## OPERATOR MATERIALS

[Table t15-jetem-9-2-s1]

**Diagnosis:**

Septic Abortion Complicated by Disseminated Intravascular Coagulation

**Disposition:**

OR, then the ICU

## DEBRIEFING AND EVALUATION PEARLS

### Septic Abortion

- Definition: a spontaneous or induced abortion complicated by a uterine infection. Typically, this refers to an infected placenta or uterus in the setting of pregnancy loss or removal (either surgically or medically) that occurs prior to 20 weeks of gestation. Past this, it is known as an intra-amniotic infection.^1^,^2^
- Mortality rate: There is a wide variation in reported mortality, likely due to under-reporting related to illegal abortion in certain countries. Still, mortality is estimated to be approximately 20–50% worldwide although lower in countries with legal abortion.^1^–^2^ While the death ratio between spontaneous and induced abortion is approximately equal in developed countries, other factors like poor maternal health and advanced gestational age are associated with higher mortality. 1
- Clinical presentation: a combination of any of the following symptoms including pelvic pain, abdominal pain, fever, uterine tenderness, purulent vaginal discharge or bleeding.^3^ Depending on the severity of the infection, patients can present with a range of mild symptoms to toxicity.
- Diagnosis: primarily clinical, based on history and exam.^3^ Clinicians should have a low threshold to work up a patient for this given the prevalence of spontaneous abortions in females who may not have known they were pregnant. Transabdominal or transvaginal ultrasound can be useful to confirm pregnancy loss and/or assess for retained products of conception but is not required for diagnosis.^3^
  ○ Differential diagnoses (including but not limited to):
    ▪ Lower abdominal pain in women of child-bearing age: ureteral colic, appendicitis, pelvic inflammatory disease, tubo-ovarian abscess, cystitis, ectopic pregnancy, endometriosis, ruptured ovarian cyst, ovarian torsion, endometritis
    ▪ Lower abdominal pain as a pregnancy or post-partum related complication: ectopic, placental abruption, uterine rupture, retained POC, endometritis
- Work-up: should include blood cultures, sexually transmitted infection screening, beta-hcg, UA and urine culture, complete blood count, complete metabolic panel, lactate level, coagulation factors, blood type and screen.^1^,^2^,^4^ If the patient is stable, you may consider performing a pelvic ultrasound to evaluate for products of conception, intrauterine pregnancy, evidence of tubo-ovarian abscess or other source of infection prior to obstetrics consultation though this is not necessary for diagnosis.
  ○ Differential diagnoses (including but not limited to):
    ▪ Anemia and thrombocytopenia: cirrhosis, DIC, Hemolysis/Elevated Liver enzymes/Low Platelets (HELLP syndrome) [second half of pregnancy or immediately postpartum], myelodysplastic syndrome, thrombotic thrombocytopenic purpura/hemolytic uremic syndrome, heparin-induced thrombocytopenia
- Management: is largely dependent on the stability of the patient. In cases of unstable patients, it is prudent to assume septic abortion in the setting of a story/exam that fits and obtain very early obstetrics consultation for source control via dilation and curettage which is definitive management.^3^ In some cases, ex-laparotomy is indicated if there is evidence of associated uterine perforation, gas, or tissue necrosis. At the same time, begin emergent aggressive resuscitation following typical sepsis guidelines involving a 30cc/kg fluid bolus within the first three hours of presentation and/or vasopressors. If there is concomitant disseminated intravascular coagulation with associated hemorrhage, resuscitation should include blood products such as packed red blood cells, platelets and cryoprecipitate as indicated (see below for more information regarding this).^4^,^5^
- Antibiotic choice: In all patients, antibiotics should be initiated early with full coverage of polymicrobial infections including urogenital flora, gastrointestinal flora, or toxin-producing bacteria. First line combination is typically IV ampicillin 2 grams IV every four hours, gentamicin 5 mg/kg/day, and the addition of either metronidazole 500mg IV every eight hours or clindamycin 900mg IV every eight hours.
- Single agent regiments include imipenem-cilastatin, meropenem, or piperacillin-tazobactam. 1,2
- Consider adding clindamycin in patients with concern of necrotizing infections or those who do not respond to initial therapy.
- Continue antibiotics for 10–14 days in some form as indicated based on culture data/clinical response.^2^

### Disseminated Intravascular Coagulation (DIC)

- DIC is a complicated and dynamic process involving accelerated formation of microthrombi and increased risk of bleeding due to depletion of coagulation factors.^6^
  ○ Chronic DIC is when there is appropriate physiologic compensation between bleeding and clotting processes in a chronically ill patient.
  ○ Acute DIC is a medical emergency that may present as a mixed picture of ischemia related to excess clotting and hemorrhagic pathology leading to epistaxis, hematemesis, hematuria, venipuncture or mucosal bleeding, petechia or purpura.^6^,^7^
- Obstetrical causes such as HELLP syndrome, abruptio placentae, septic abortion, retained fetal parts have a higher likelihood of leading to DIC.^4^,^6^ Non-obstetrical causes include sepsis, malignancy, trauma, and surgery.
- Labs supporting DIC diagnosis includes: elevated PT/PTT, prolonged bleeding time, elevated d-dimer, and low fibrinogen.^4^,^7^ There is no one lab test to definitively diagnose DIC in isolation.
- Differential diagnoses include idiopathic thrombocytopenia, thrombotic thrombocytopenic purpura, hemolytic uremic syndrome, liver failure, vitamin K deficiency.
- Treat DIC by treating the underlying etiology.^6^ Examples of underlying etiologies include but are not limited to: trauma, obstetrical complication, intravascular hemolysis, ABO incompatibility, heat stroke, crush injuries, amphetamine overdose, fat embolism, crotalid bite, and organ rejection.
- Adjunctive therapies for DIC management are summarized in the table below.^8^,^9^ In addition, consider giving cryoprecipitate if fibrinogen is severely low <100mg/dL or <150mg/dL in a pregnant patient.^4^,^6^,^8^,^9^
- As in all cases of hemorrhage, it is imperative to avoid hypothermia and acidosis to not further exacerbate coagulopathies.^9^

### Massive Transfusion Protocol

- Generally a 1:1:1 protocol for FFP: Platelets: pRBC is used in order to promote balanced resuscitation
- Consider the addition of calcium[Table t16-jetem-9-2-s1]

**Closed Loop Communication**

▪ Pointed roles during a critical event are extremely important in providing maximal care
▪ Address team members by their roles or names when asking for an action and request a verbal confirmation that you were heard and understood

## SIMULATION ASSESSMENT

*Septic Abortion Complicated by Disseminated Intravascular Coagulation*

Learner: _________________________________________

***Assessment Timeline***

This timeline is to help observers assess their learners. It allows observer to make notes on when learners performed various tasks, which can help guide debriefing discussion.[Table t17-jetem-9-2-s1]

**Critical Actions:**

□ Place patient on monitor and obtain a full set of vitals, including temperature
□ Place two large bore IV catheters
□ Initiate administration of 30 cc/kg intravenous fluid bolus within three minutes of noting tachycardia
□ Perform a physical exam specifically including a pelvic exam
□ Order broad-spectrum antibiotics for appropriate polymicrobial coverage
□ Communicate need for D&C for retained products of conception to OB-GYN
□ Reassess patient when nursing staff notes bleeding from peripheral IV sites
□ Order a d-dimer, fibrinogen, and coagulation factors
□ Verbalize concern for DIC
□ Administer packed red blood cells, fresh frozen plasma, and platelets in a 1:1:1 ratio.
□ Admit to the ICU for further management of sepsis and bleeding

**Summative and formative comments:**

**Milestones assessment:** [Table t18-jetem-9-2-s1]
