# Supplementary material for: Septic Abortion Complicated by Disseminated Intravascular Coagulation
Source: J Educ Teach Emerg Med. 2024 Apr 30;9(2):S1–S26. doi: 10.21980/J8GH1G (PMC11068314; doi:10.21980/J8GH1G)

## Slide 1
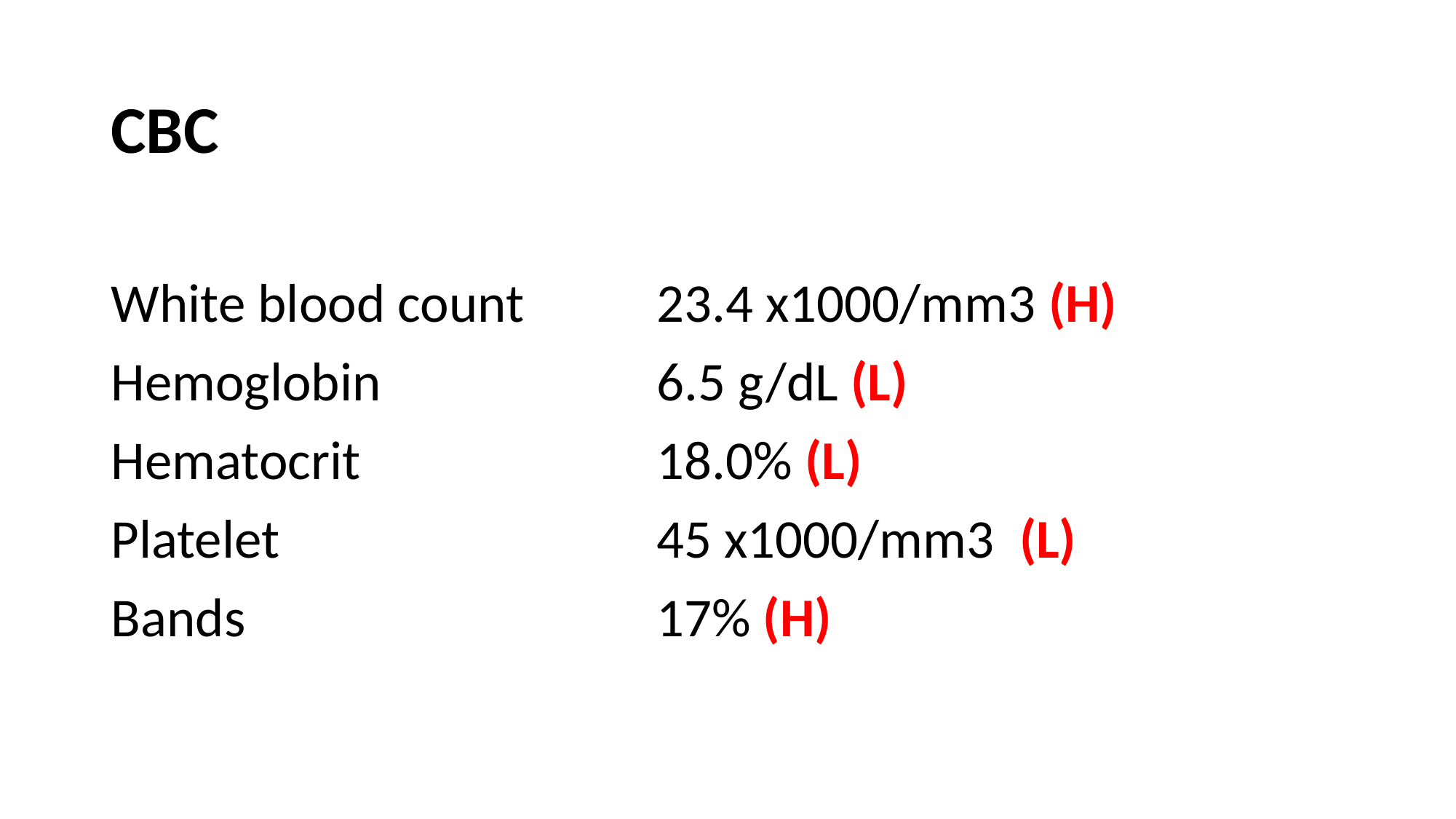

CBC
White blood count 	23.4 x1000/mm3 (H)
Hemoglobin 	 		6.5 g/dL (L)
Hematocrit 			18.0% (L)
Platelet 	 			45 x1000/mm3 (L)
Bands 				17% (H)

## Slide 2
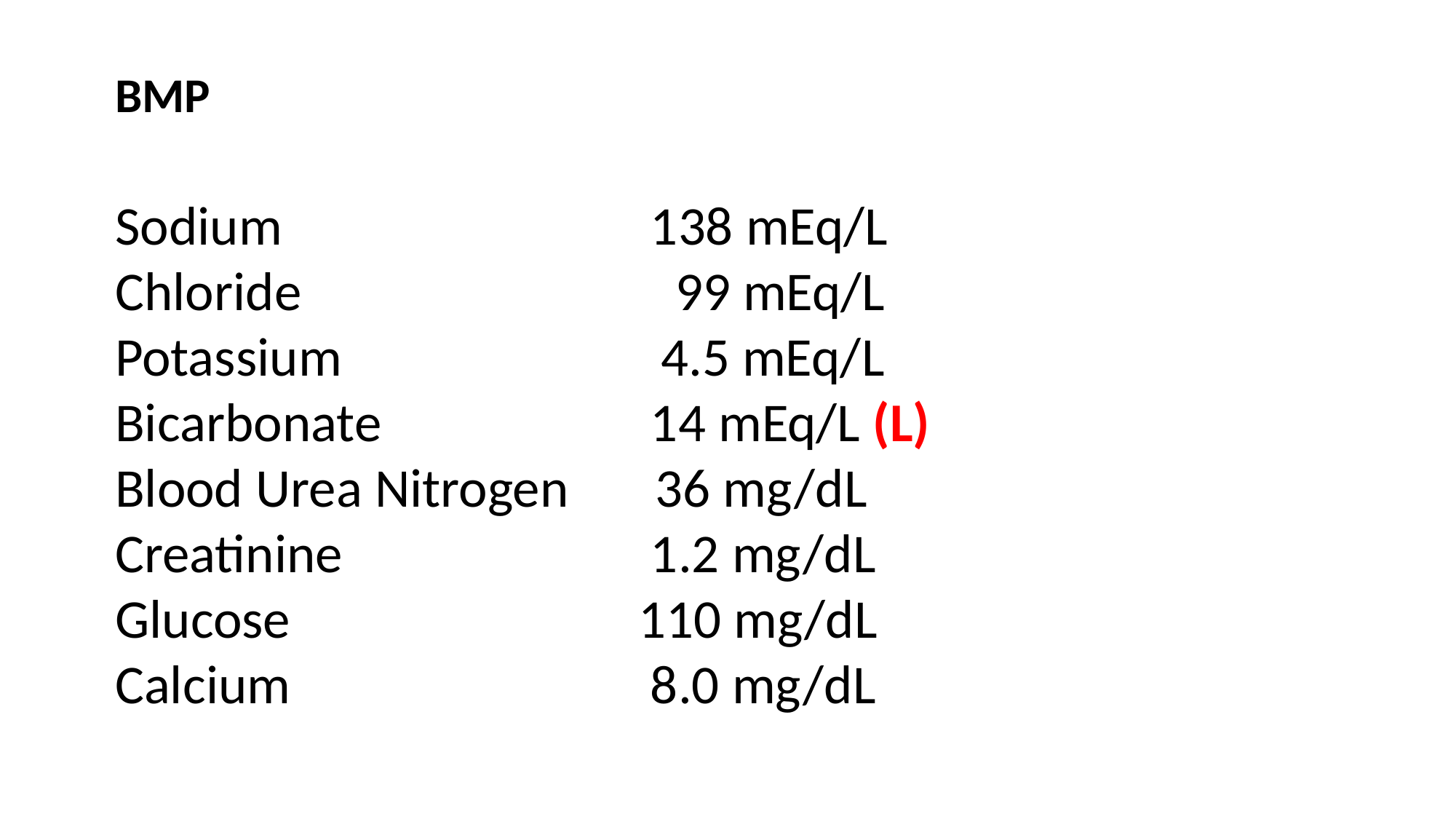

BMP
Sodium 		 138 mEq/L
Chloride 		 99 mEq/L
Potassium 			4.5 mEq/L
Bicarbonate 		 14 mEq/L (L)
Blood Urea Nitrogen 36 mg/dL
Creatinine 		 1.2 mg/dL
Glucose 		 110 mg/dL
Calcium 			 8.0 mg/dL

## Slide 3
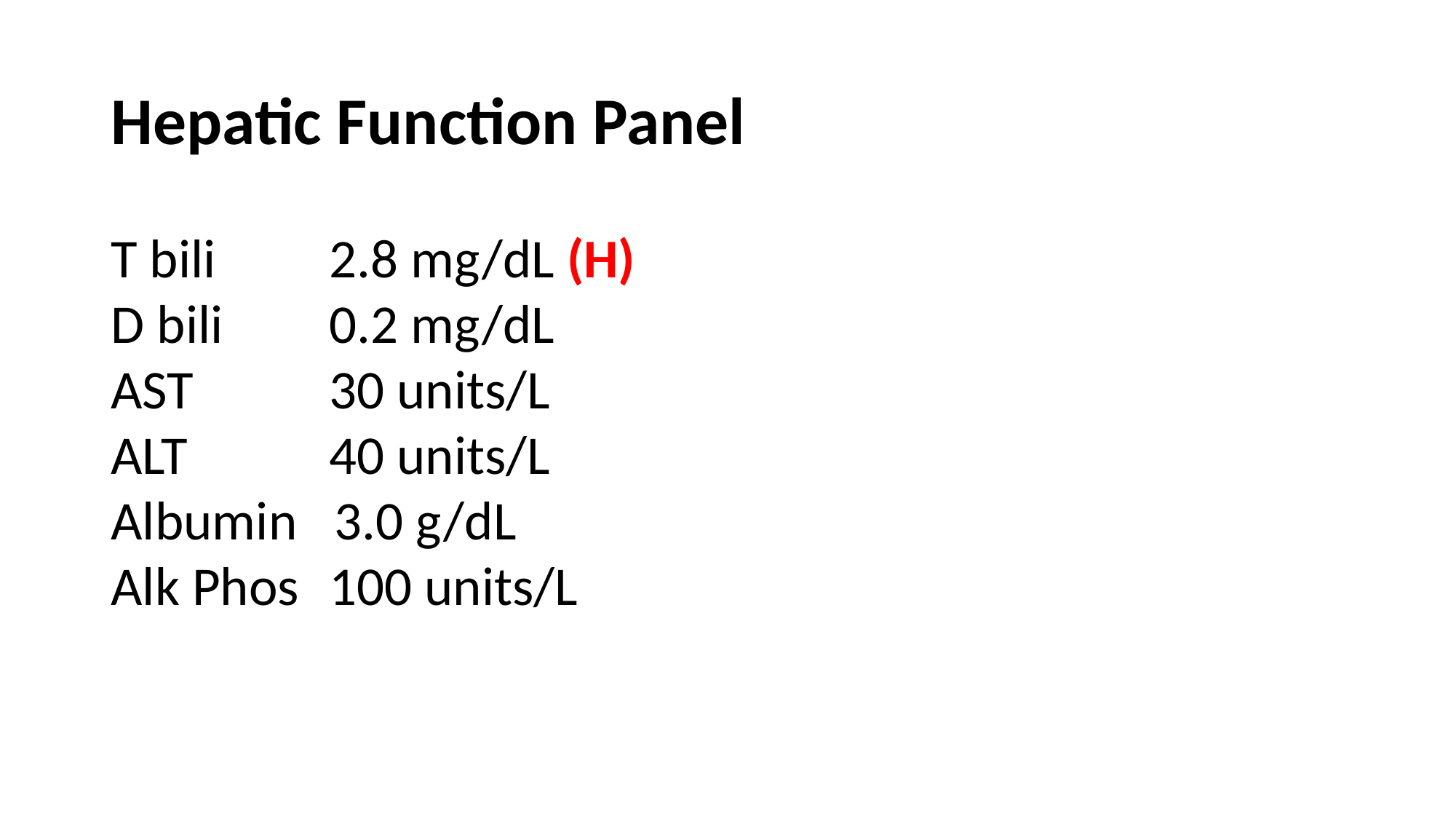

# Hepatic Function Panel
T bili 	2.8 mg/dL (H)
D bili 	0.2 mg/dL
AST 		30 units/L
ALT 		40 units/L
Albumin 3.0 g/dL
Alk Phos 	100 units/L

## Slide 4
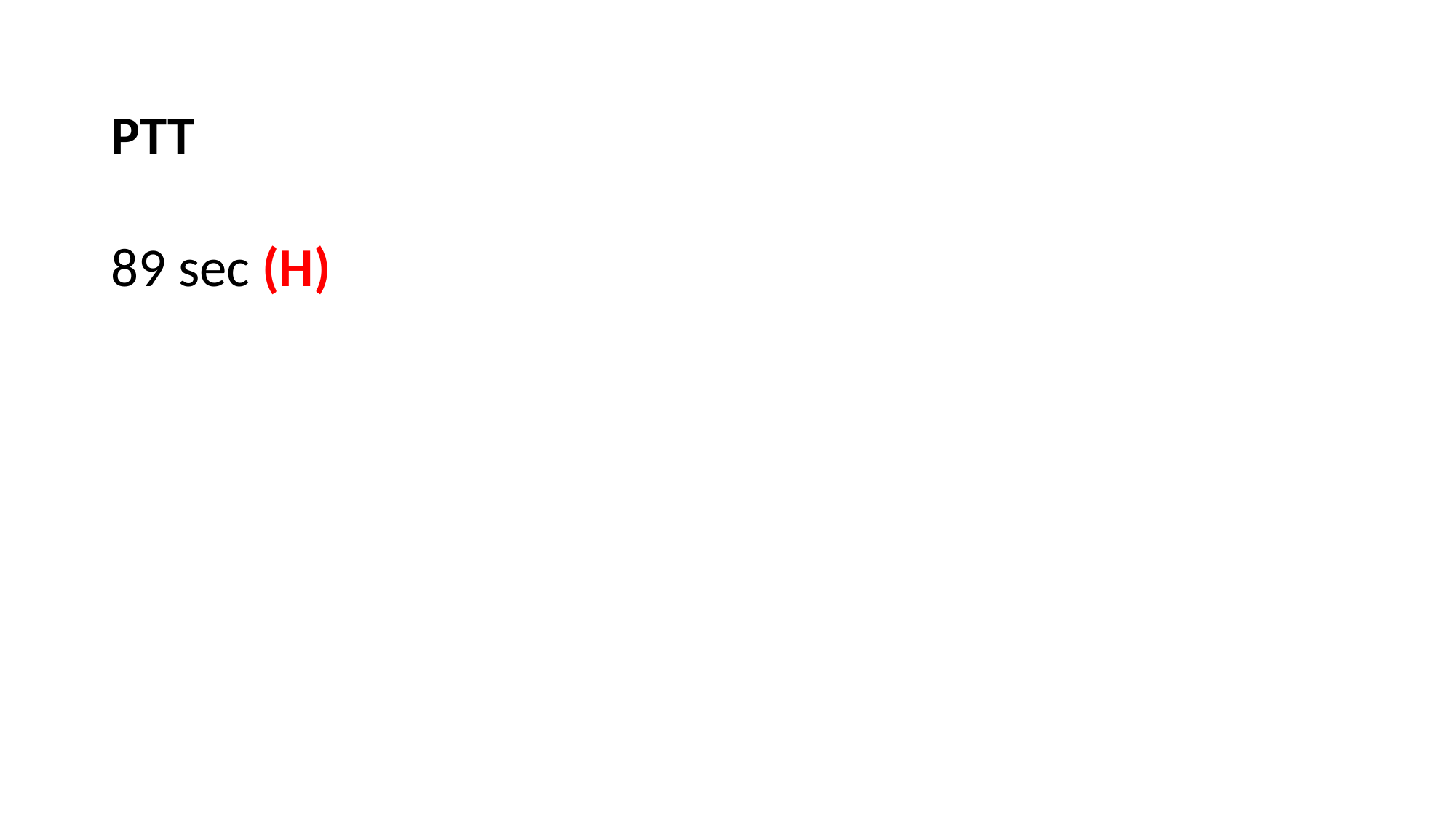

PTT
89 sec (H)

## Slide 5
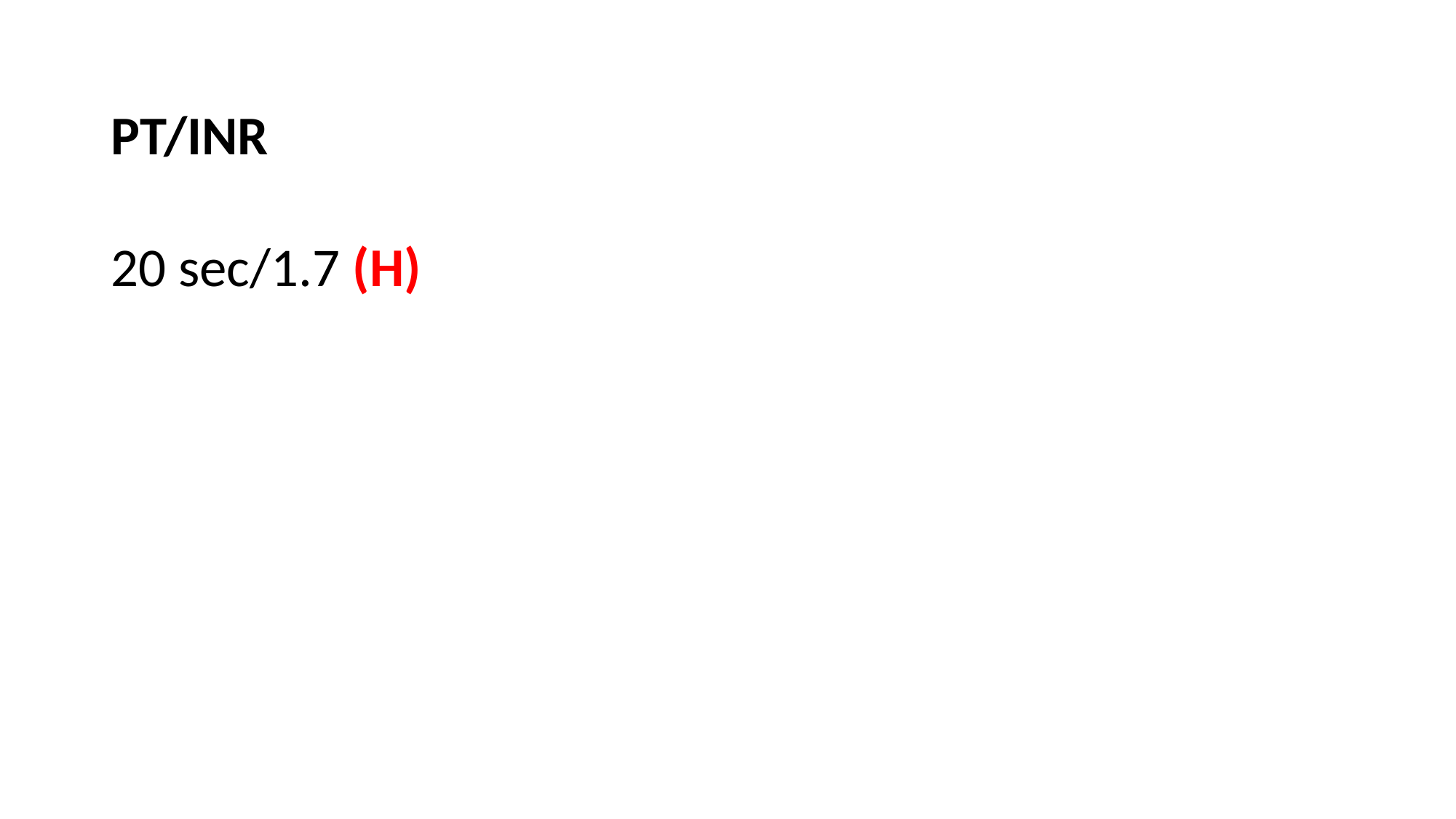

PT/INR
20 sec/1.7 (H)

## Slide 6
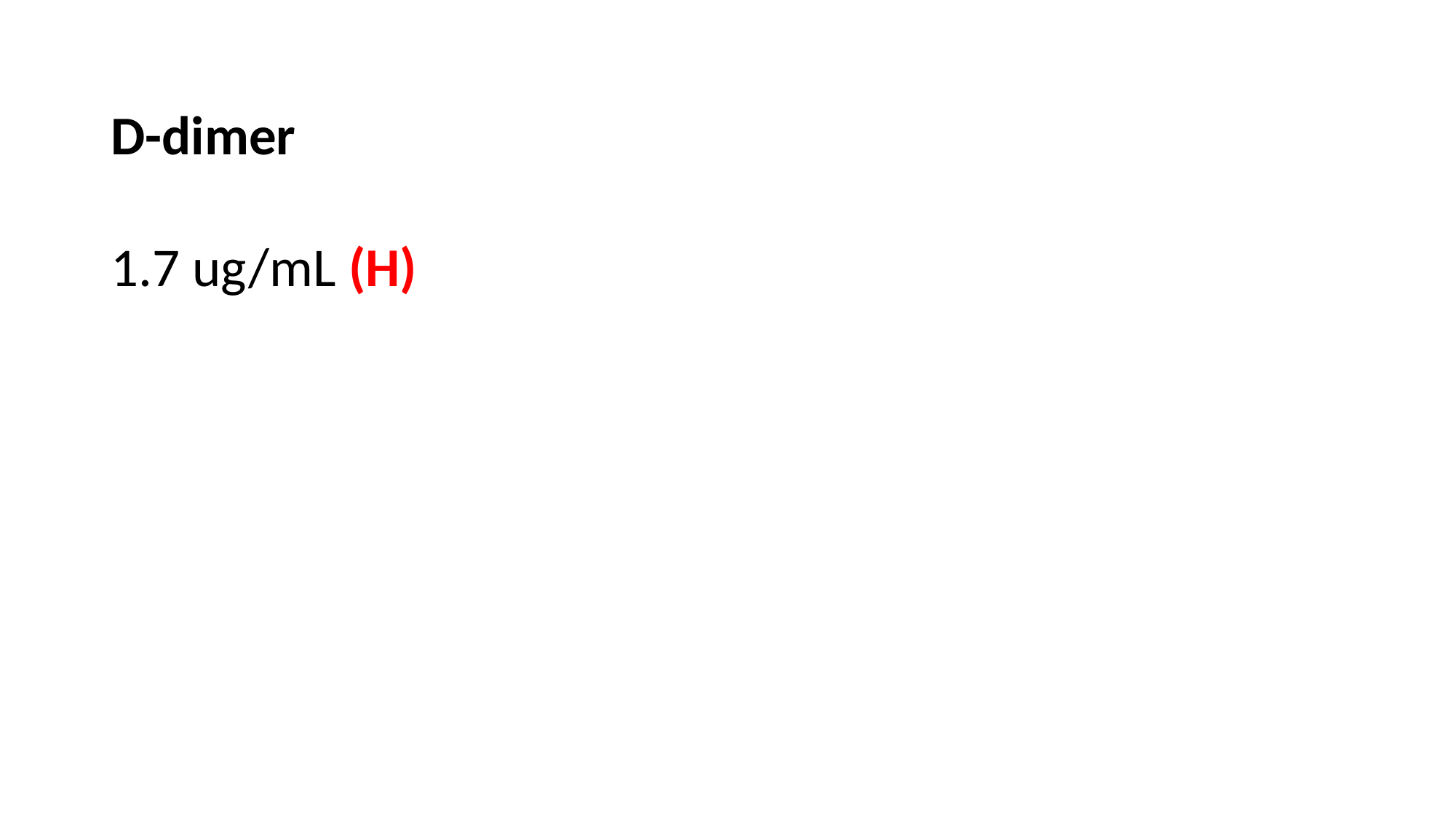

D-dimer
1.7 ug/mL (H)

## Slide 7
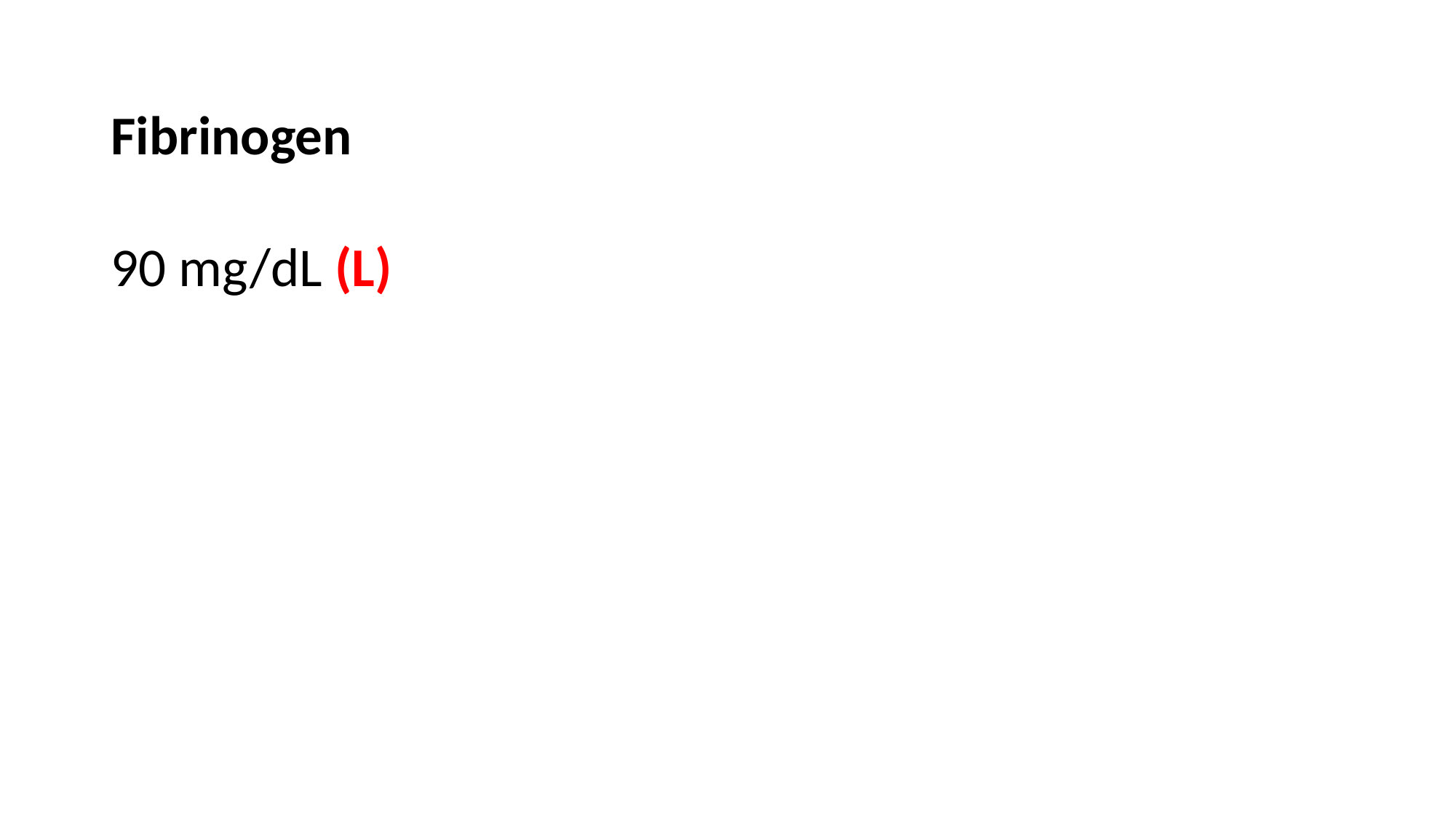

Fibrinogen
90 mg/dL (L)

## Slide 8
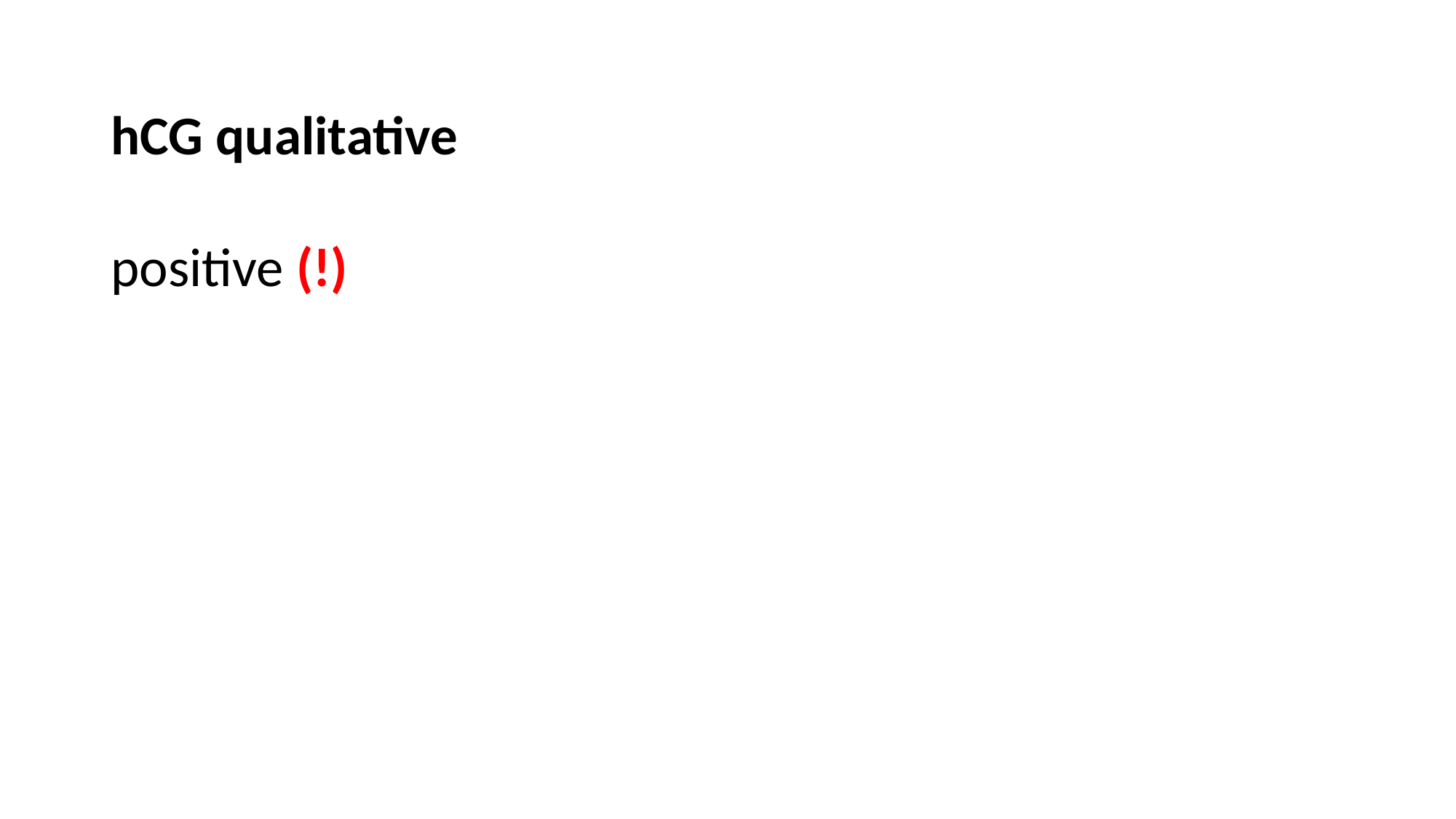

hCG qualitative
positive (!)

## Slide 9
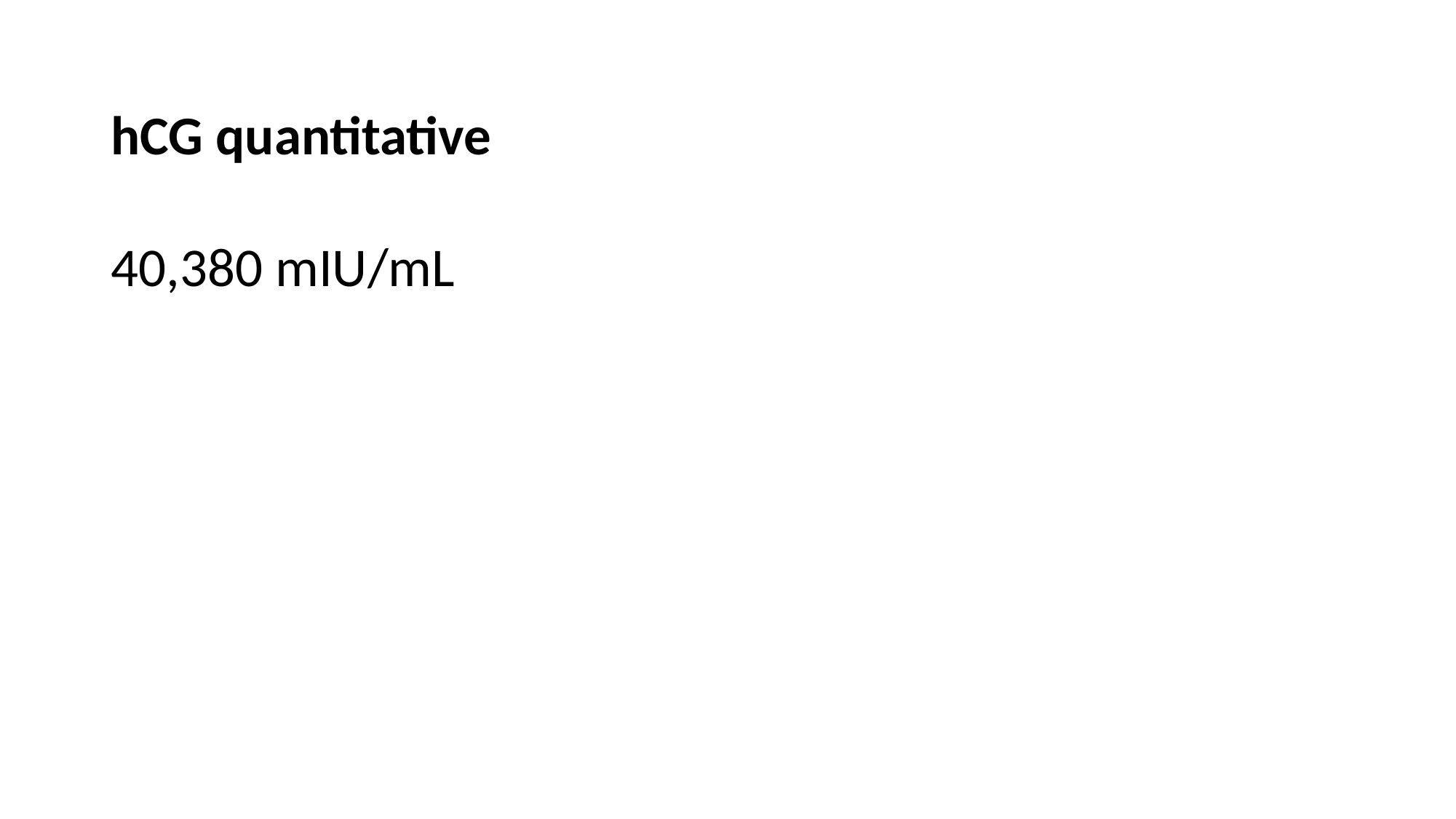

hCG quantitative
40,380 mIU/mL

## Slide 10
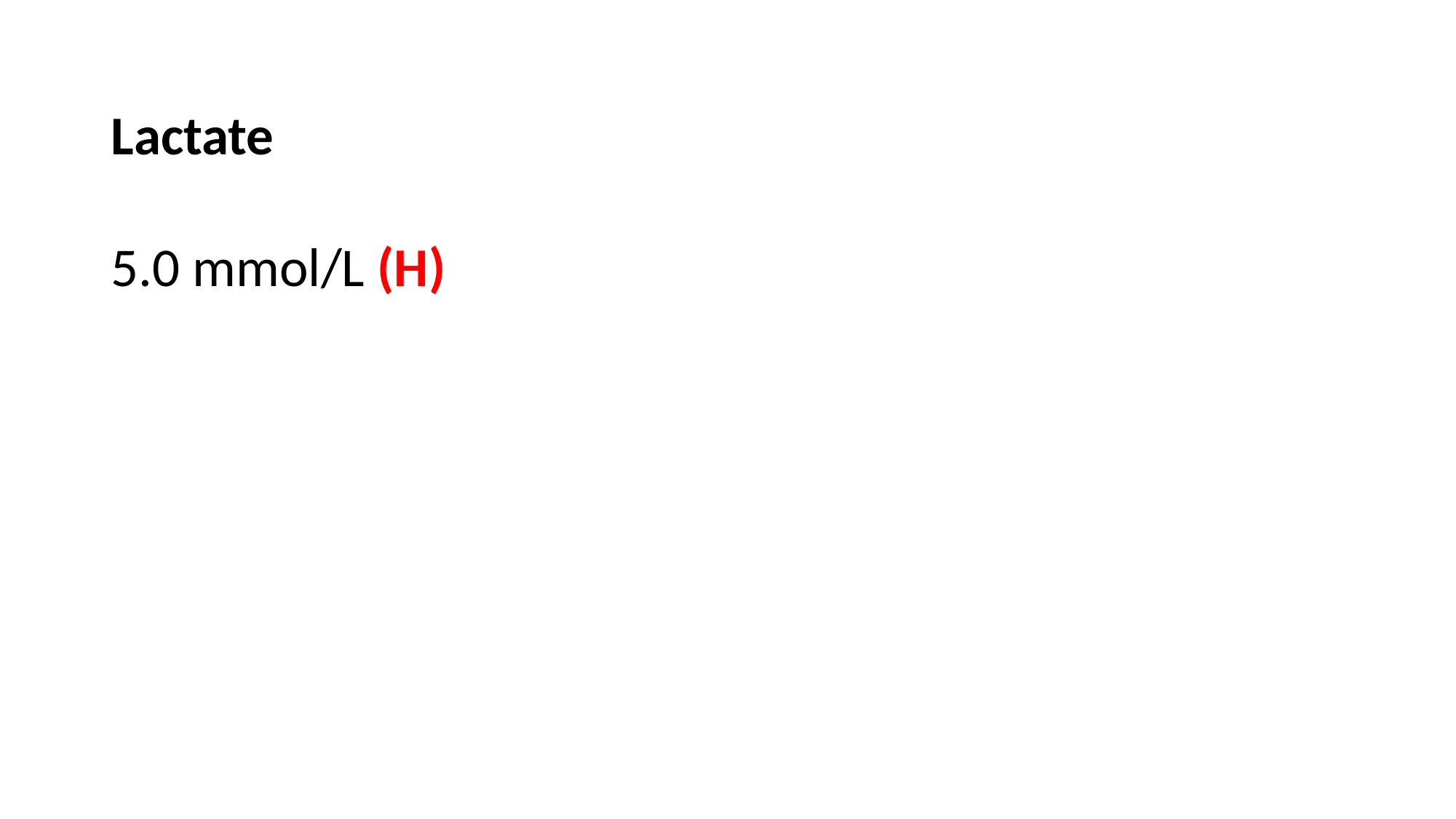

Lactate
5.0 mmol/L (H)

## Slide 11
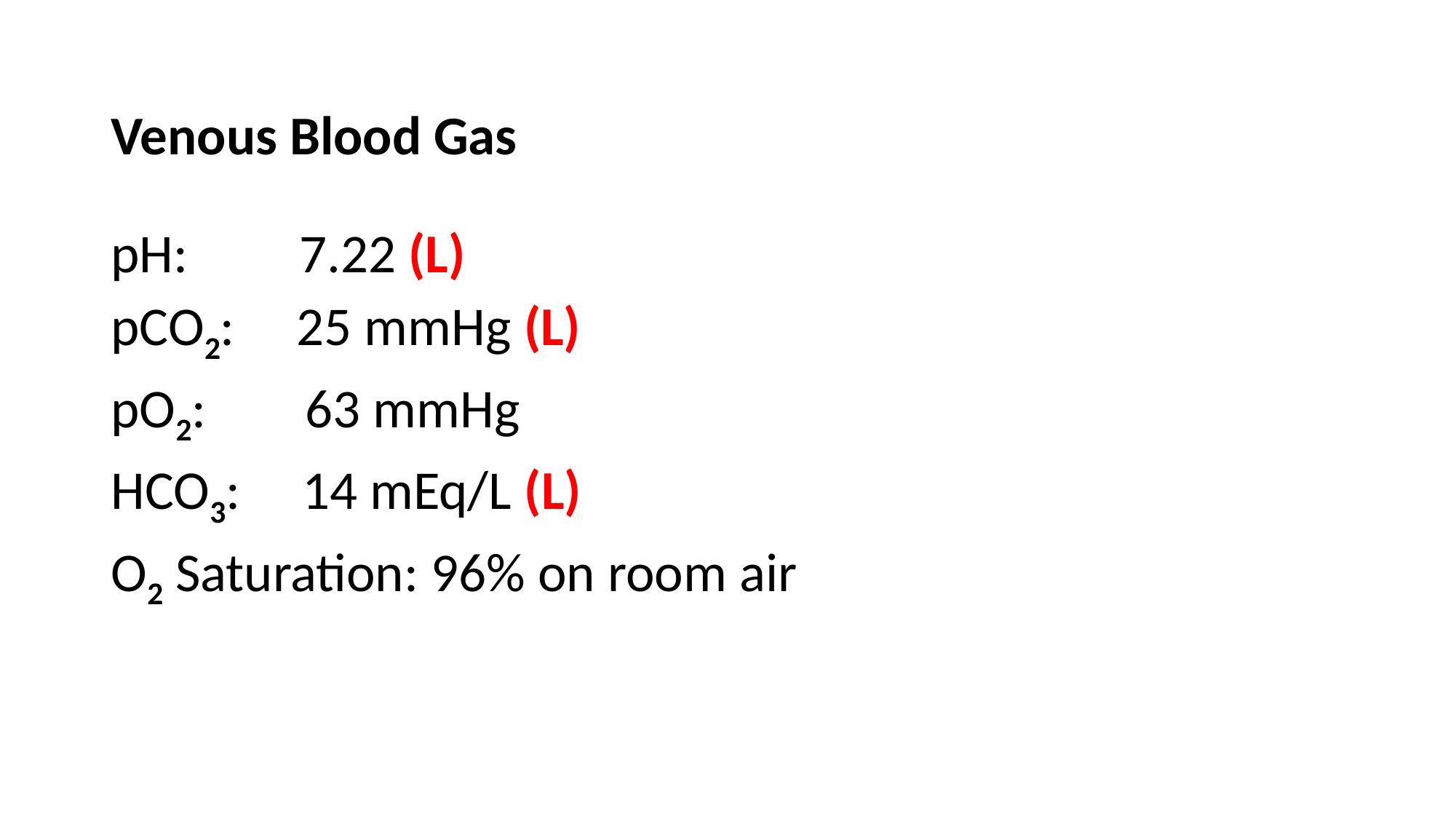

Venous Blood Gas
pH: 7.22 (L)
pCO2: 25 mmHg (L)
pO2: 63 mmHg
HCO3: 14 mEq/L (L)
O2 Saturation: 96% on room air

## Slide 12
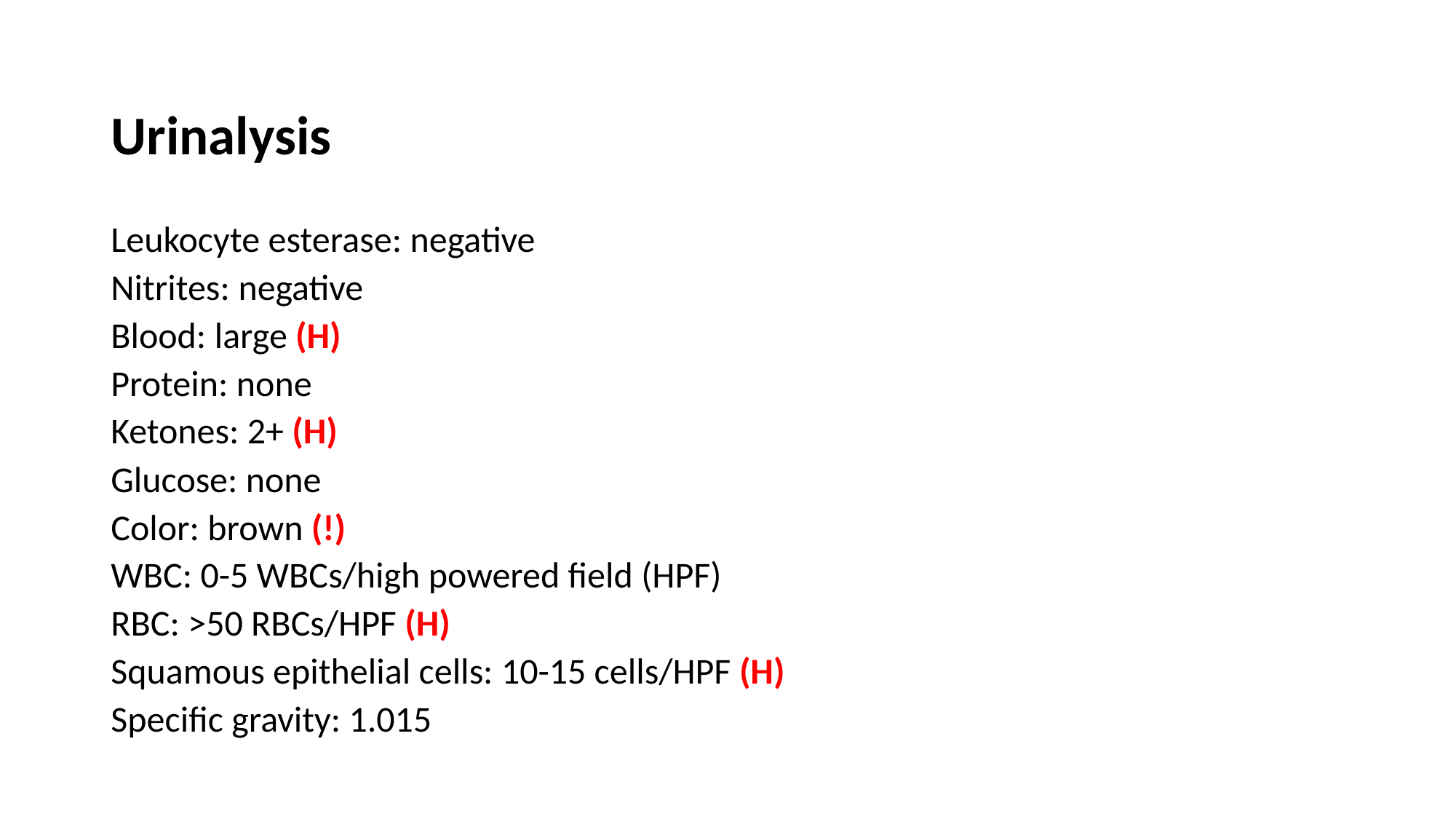

Urinalysis
Leukocyte esterase: negative
Nitrites: negative
Blood: large (H)
Protein: none
Ketones: 2+ (H)
Glucose: none
Color: brown (!)
WBC: 0-5 WBCs/high powered field (HPF)
RBC: >50 RBCs/HPF (H)
Squamous epithelial cells: 10-15 cells/HPF (H)
Specific gravity: 1.015

## Slide 13
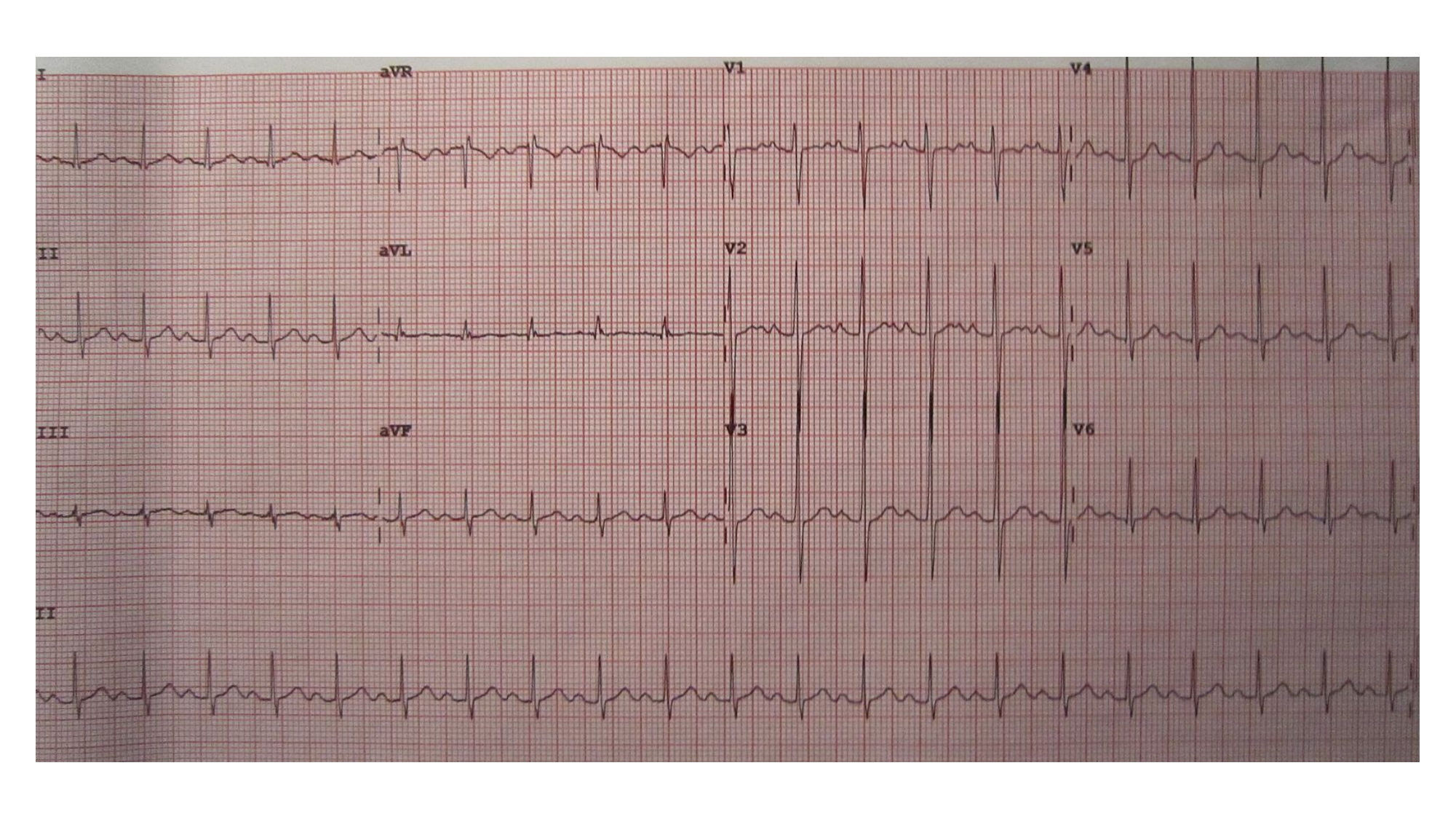

## Slide 14
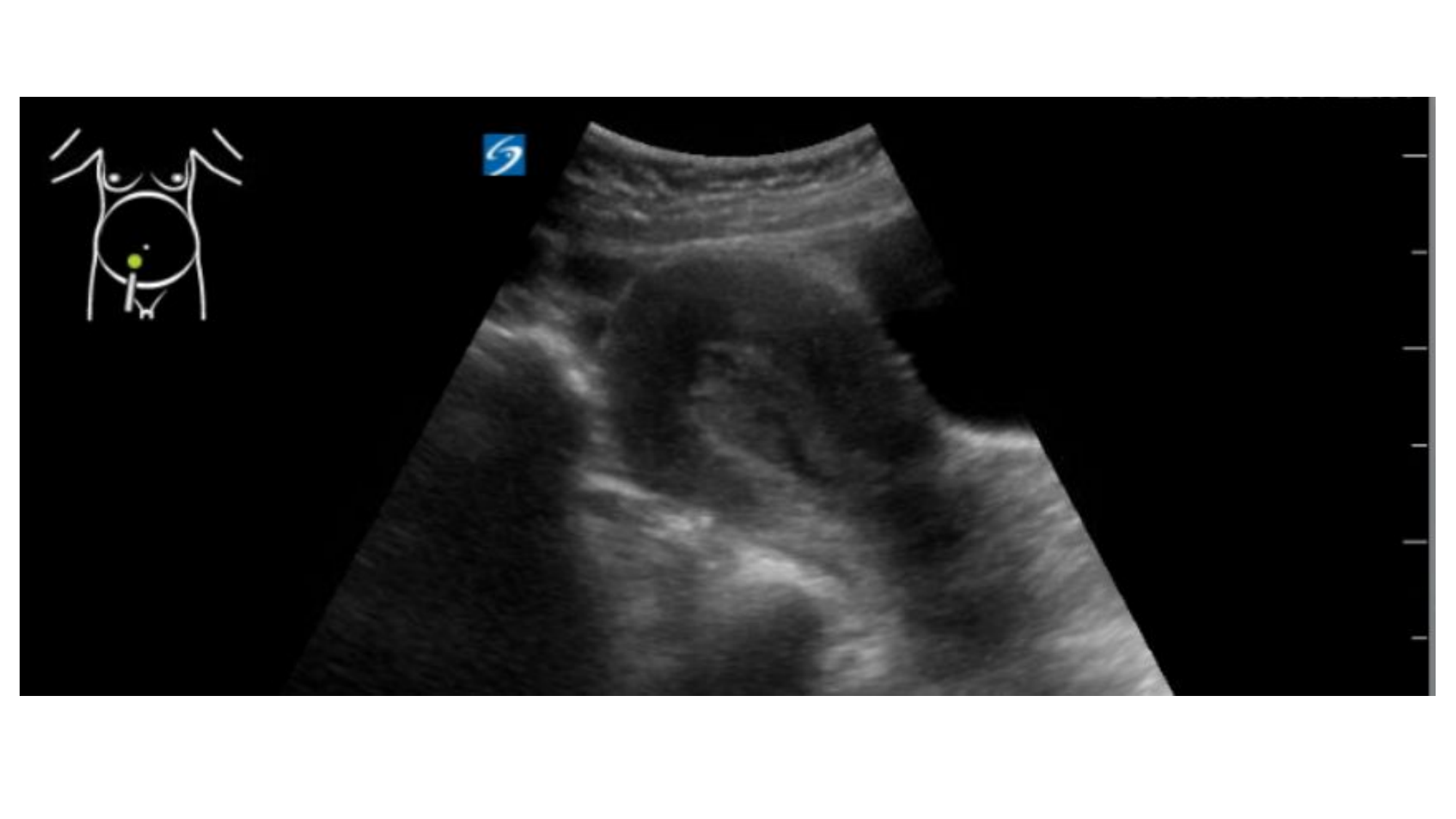

## Slide 15
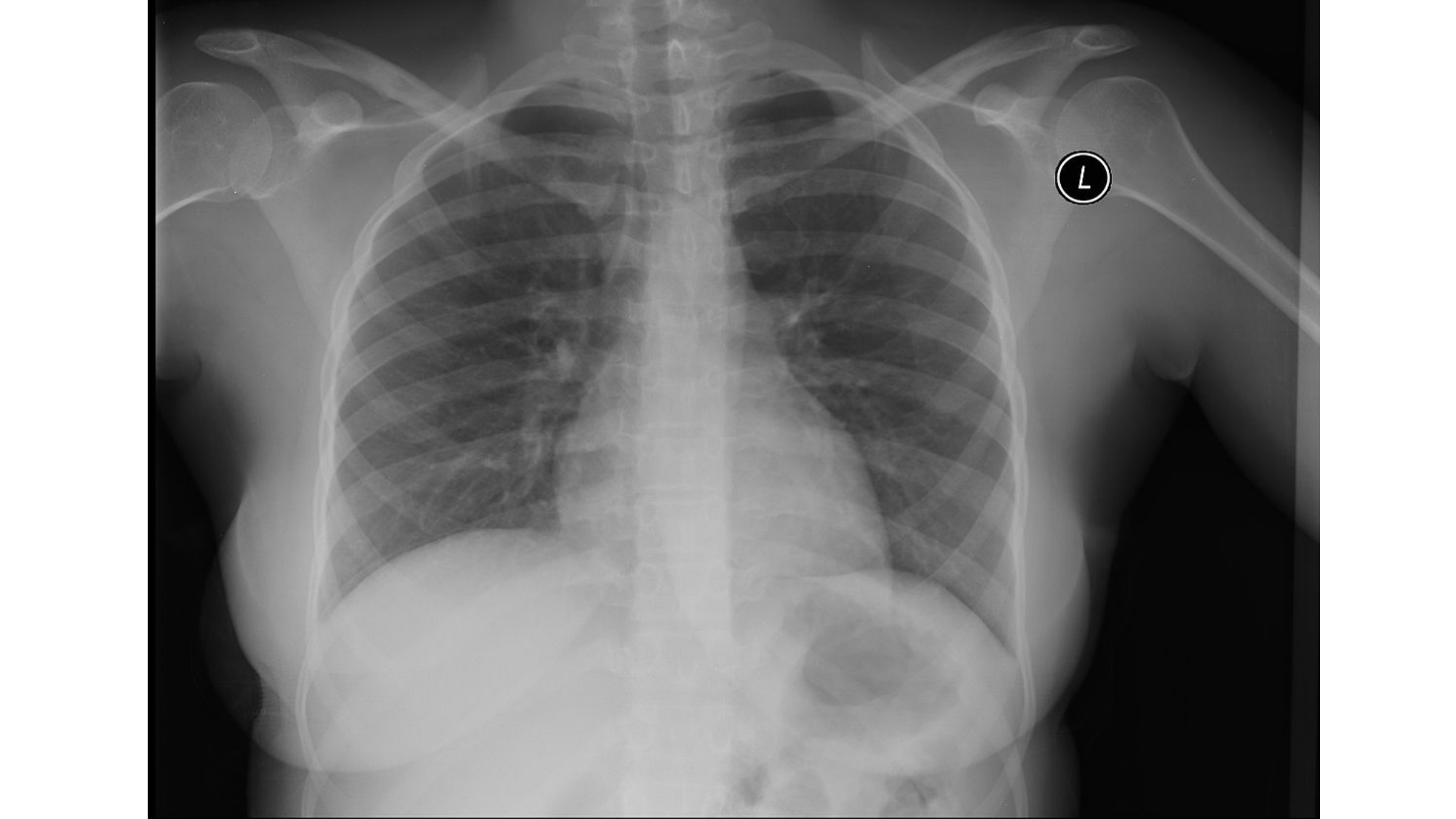

Supplement: Supplementary file 1 [file jetem-9-2-S1-supp1.pptx]
